# Mbf1 ensures Polycomb silencing by protecting *E(z)* mRNA from degradation by Pacman

**DOI:** 10.1242/dev.162461

**Published:** 2018-03-01

**Authors:** Kenichi Nishioka, Xian-Feng Wang, Hitomi Miyazaki, Hidenobu Soejima, Susumu Hirose

**Affiliations:** 1 Division of Molecular Genetics and Epigenetics, Department of Biomolecular Sciences, Faculty of Medicine, Saga University, 5-1-1 Nabeshima, Saga City, Saga 849-8501, Japan; 2 Division of Gene Expression, Department of Developmental Genetics, National Institute of Genetics, 1111 Yata, Mishima City, Shizuoka 411-8540, Japan

**Keywords:** Mbf1, Enhancer of zeste, E(z), Polycomb silencing, Pcm, Exoribonuclease, *Drosophila melanogaster*

## Abstract

Under stress conditions, the coactivator Multiprotein bridging factor 1 (Mbf1) translocates from the cytoplasm into the nucleus to induce stress-response genes. However, its role in the cytoplasm, where it is mainly located, has remained elusive. Here, we show that *Drosophila* Mbf1 associates with *E(z)* mRNA and protects it from degradation by the exoribonuclease Pacman (Pcm), thereby ensuring Polycomb silencing. In genetic studies, loss of *mbf1* function enhanced a Polycomb phenotype in Polycomb group mutants, and was accompanied by a significant reduction in *E(z)* mRNA expression. Furthermore, a *pcm* mutation suppressed the Polycomb phenotype and restored the expression level of *E(z)* mRNA, while *pcm* overexpression exhibited the Polycomb phenotype in the *mbf1* mutant but not in the wild-type background. *In vitro*, Mbf1 protected *E(z)* RNA from Pcm activity. Our results suggest that Mbf1 buffers fluctuations in Pcm activity to maintain an *E(z)* mRNA expression level sufficient for Polycomb silencing.

## Abstract (precis)

**Highlighted Article:** In addition to its role as a nuclear coactivator, a cytoplasmic mRNA-stabilizing function of Multiprotein bridging factor 1 may contribute to various types of stress defense, metabolic processes and neurogenesis in *Drosophila*.

## INTRODUCTION

Polycomb silencing is essential for the developmental regulation of gene expression (Grossniklaus and Paro, 2014; Comet et al., 2016; Kundu et al., 2017). The silencing needs to be robust to tightly repress the expression of developmental genes in undifferentiated cells, such as stem cells, but should also be flexible for rapid release upon differentiation. However, this paradoxical aspect of Polycomb silencing is not well understood.

Mbf1 was originally identified as an evolutionarily conserved coactivator that connects a transcriptional activator with the TATA element-binding protein (Li et al., 1994; Takemaru et al., 1997, 1998). Usually, Mbf1 is present in the cytoplasm; however, under stress conditions, Mbf1 translocates into the nucleus to induce stress-response genes (Kabe et al., 1999; Jindra et al., 2004; Ballabio et al., 2004). Previous studies have revealed roles for the coactivator in axon guidance (Liu et al., 2003), oxidative stress response (Jindra et al., 2004; Arce et al., 2010), heat-shock response (Suzuki et al., 2008), defense against microbial infection (Suzuki et al., 2005; Kim et al., 2007), and resistance to drugs such as tamoxifen (Mendes-Pereira et al., 2012). However, the cytoplasmic role of Mbf1 has remained elusive, except for mRNA or ribosomal binding (Baltz et al., 2012; Klass et al., 2013; Kwon et al., 2013; Blombach et al., 2014).

Pacman (Pcm/Xrn1) is an evolutionarily conserved 5′-3′ exoribonuclease that degrades decapped mRNA (Till et al., 1998; Jones et al., 2012). Genetic studies have demonstrated that *Drosophila pcm* is involved in epithelial closure, male fertility, apoptosis and growth control (Grima et al., 2008; Lim et al., 2009; Jones et al., 2012, 2016; Waldron et al., 2015). Null mutants of *pcm* are lethal during early pupal stages, suggesting the enzyme plays an essential role in development (Waldron et al., 2015; Jones et al., 2016).

Using a genetic approach in *Drosophila*, we show that cytoplasmic Mbf1 ensures Polycomb silencing by protecting *E(z)* mRNA from degradation by Pcm. Our results thus demonstrate an unexpected component of the regulatory mechanism underlying Polycomb silencing. This mechanism might also allow flexibility in Polycomb silencing, as Mbf1 protein expression declines upon differentiation.

## RESULTS AND DISCUSSION

### Mbf1 enhances Polycomb silencing by protecting *E(z)* mRNA in the cytoplasm

**Fig. 1.**
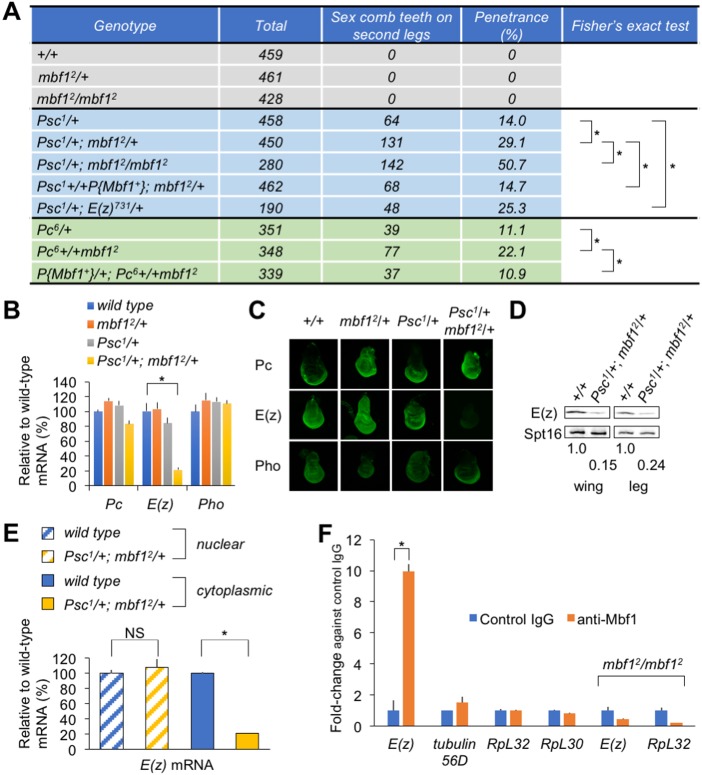
**Compromised *E(z)* expression in the *mbf1* mutant in a Polycomb group mutant background.** (A) Genetic interactions between *mbf1* and Polycomb group mutants. The P-element vector *P{mbf1^+^}* expresses wild-type Mbf1 from a transgene. *P<0.01 (Fisher's exact test). (B) RT-qPCR analysis of the indicated Polycomb group mRNAs in whole extracts from third instar male larvae. Data are mean±s.d., relative to the wild-type mRNA level; *P<0.01 (Student's *t*-test). (C) Immunofluorescence analyses of indicated Polycomb group proteins in wing discs of third instar larvae. (D) Western blot analyses of E(z) in wing or leg discs. Numbers indicate relative E(z) levels normalized to those of Spt16. (E) RT-qPCR analysis of *E(z)* mRNA in the nuclear or cytoplasmic fraction of wing discs. NS, not significant; *P<0.01 (Student's *t*-test). (F) Mbf1 binds to *E(z)* mRNA. RIP samples from wild-type or *mbf1^2^* embryonic extracts were analyzed by RT-qPCR. Data are mean±s.d. of fold-change versus control IgG; *P<0.01 (Student's *t*-test).

To address the cytoplasmic role of Mbf1, we searched for novel genes that interact with *mbf1*. Surprisingly, the *mbf1* mutation enhanced a classical Polycomb phenotype of *Psc* and *Pc* mutants, namely the appearance of an ectopic sex comb tooth or teeth on the male mid-leg (Fig. 1A). Although *mbf1^2^*/*+* or *mbf1^2^*/*mbf1^2^* flies never exhibited the Polycomb phenotype, penetrance of the phenotype in *Psc^1^*/*+* increased significantly in *Psc^1^*/*+*; *mbf1^2^*/*+*, and further increased in *Psc^1^*/*+*; *mbf1^2^*/*mbf1^2^*. The penetrance was restored to the *Psc^1^*/*+* level by expressing wild-type Mbf1 protein from a transgene. Similar effects of the *mbf1^2^* allele were observed with the *Pc^6^* mutation.

To gain insight into the mechanism underlying the genetic interaction between *Psc* and *mbf1*, we examined the expression of the representative Polycomb group genes *Pc*, *E(z)* and *pho*. Results of reverse transcription-quantitative PCR (RT-qPCR) analyses demonstrated a prominent reduction in the expression level of *E(z)* mRNA in *Psc^1^*/*+*; *mbf1^2^*/*+* larvae, whereas *Pc* and *pho* mRNA levels remained unchanged (Fig. 1B). Immunostaining of wing discs demonstrated that E(z) protein expression was severely compromised in *Psc^1^*/*+*; *mbf1^2^*/*+* compared with that in wild type, *mbf1^2^*/*+* or *Psc^1^/+* (Fig. 1C). By contrast, the expression of Pc and Pho proteins was not significantly affected. Western blot analyses confirmed the marked decrease in the E(z) protein level in both wing and leg discs from *Psc^1^*/*+*; *mbf1^2^*/*+* (Fig. 1D). Consistently, *Psc^1^*/*+*; *E(z)^731^/+* exhibited the extra sex comb phenotype, which was comparable to *Psc^1^*/*+*; *mbf1^2^*/*+* (Fig. 1A).

It is unlikely that Mbf1 affects *E(z)* transcription because no significant difference was detected in the *E(z)* mRNA level between wild-type and *mbf1^2^*/*mbf1^2^* larvae (Fig. S1A). Consistently, we were unable to detect any significant difference in the expression of E(z) in the wing disc upon knockdown or overexpression of Mbf1 using a posterior compartment-specific Gal4 driver (Fig. S1B). When cytoplasmic and nuclear RNA fractions from wing discs were analyzed by RT-qPCR, the nuclear *E(z)* mRNA level was similar between wild type and *Psc^1^*/*+*; *mbf1^2^*/*+*. However, the cytoplasmic *E(z)* mRNA level in *Psc^1^*/*+*; *mbf1^2^*/*+* decreased to ∼20% of the wild-type level (Fig. 1E). Collectively, these results suggest that *mbf1* regulates the *E(z)* mRNA level post-transcriptionally in the cytoplasm.

Considering that Mbf1 binds to mRNA (Baltz et al., 2012; Klass et al., 2013; Kwon et al., 2013), we hypothesized that cytoplasmic Mbf1 might bind to *E(z)* mRNA to protect it from degradation, and thereby regulates the *E(z)* mRNA level. Results of RNA-immunoprecipitation (RIP) experiments revealed a preferential binding of Mbf1 to *E(z)* mRNA. We found a ∼10-fold enrichment of *E(z)* mRNA in the anti-Mbf1 antibody pull-down fraction from cytoplasmic extracts of embryos (Fig. 1F). The pull-down was clearly selective, as enrichment of abundant mRNAs, such as *RpL32* and *RpL30*, was not observed. By contrast, *E(z)* mRNA was barely detectable in the anti-Mbf1 antibody pull-down fraction from embryonic extracts of the *mbf1* mutant, used as a negative control. This is not due to absence of *E(z)* mRNA in the *mbf1* mutant (Fig. S1A).

### Pcm counteracts Polycomb silencing

**Fig. 2.**
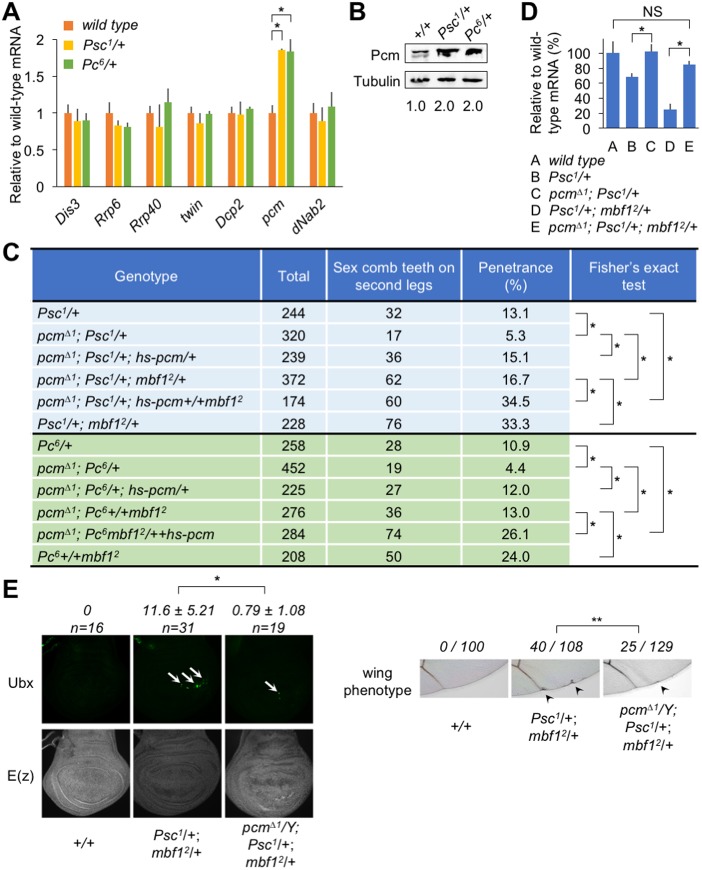
**Functional relationship among *mbf1*, *E(z)* and *pcm*.** (A) *pcm* is downregulated by *Psc* and *Pc*. Expression of the indicated genes in third instar male larvae was analyzed by RT-qPCR in the wild-type or Polycomb group mutant background. *pcm* does not appear to be a direct target of Polycomb silencing (Zeng et al., 2012). Data are mean±s.d. relative to the wild-type mRNA level; *P<0.01 (Student's *t*-test). (B) Western blot analysis of Pcm in wing discs from the indicated lines. Numbers indicate relative Pcm levels normalized to Tubulin levels. (C) *pcm* mutation suppresses the extra sex comb phenotype. *P<0.01 (Fisher's exact test). (D) *pcm* mutation restores the *E(z)* mRNA level in *Psc^1^/+* or *Psc^1^/+*; *mbf1^2^/+*. The *E(z)* mRNA levels in third instar male larvae of the indicated lines were analyzed by RT-qPCR. Data are mean±s.d. relative to the wild-type mRNA level. NS, not significant; *P<0.01 (Student's *t*-test). (E) (Top) Misexpression of Ubx in the wing disc of *Psc^1^/+*; *mbf1^2^/+* and its suppression by *pcm*^Δ*1*^. Arrows indicate Ubx-positive spots. (Bottom) Immunostaining of E(z) protein in the wing discs shown above. (Right) Adult wing defect (arrowheads) in *Psc^1^/+*; *mbf1^2^/+* and its suppression by *pcm*^Δ*1*^. The number of wings with the defect among the total number of wings examined is indicated. **P<0.05 (Fisher's exact test).

Following the observed preferential binding of Mbf1 to *E(z)* mRNA, we focused on the Polycomb phenotype and reduced *E(z)* mRNA expression level, which were not caused by the *mbf1* mutation alone. Enhancement of the Polycomb phenotype and the reduction of *E(z)* mRNA were only detected in the double *mbf1* and Polycomb group gene mutant. To explain the synergistic effect of *mbf1* and Polycomb group mutations, we posited that a component of the mRNA degradation pathway was only activated in the Polycomb group mutant background. Therefore, we sought to identify the component of the pathway that was activated in the *Psc* or *Pc* mutants. Among the mRNAs tested, only *pcm* mRNA, which encodes the 5′-exoribonuclease, was upregulated in *Psc^1^*/*+* and *Pc^6^*/*+* larvae (Fig. 2A). Neither the decapping enzyme (*Dcp2*), components of the exosome [*Dis3*, *Prp6* (*CG6841*) and *Prp40* (*CG3542*)] (Siwaszek et al., 2014), nor components in the 3′-deadenylation-mediated pathway (*twin* and *Nab2*) (Morris et al., 2005; Pak et al., 2011) appeared to be activated. Western blot analyses revealed a 2-fold increase in the Pcm protein level in wing discs from *Psc^1^*/*+* or *Pc^6^*/*+* larvae compared with that from wild type (Fig. 2B)*.* These results led us to further investigate the effects of the *pcm* mutation on Polycomb silencing and *E(z)* mRNA expression.

Strikingly, the *pcm*^Δ*1*^ mutation resulted in significant suppression of the Polycomb phenotype in *Psc^1^*/*+* and *Psc^1^*/*+*; *mbf1^2^*/*+* (Fig. 2C). This suppression was rescued by expressing the wild-type Pcm protein from a transgene. Similar results were obtained using the *Pc^6^* mutant (Fig. 2C) and another *pcm* allele, *pcm^5^* (Fig. S2). Consistent with this result, the *pcm*^Δ*1*^ mutation restored the *E(z)* mRNA levels in *Psc^1^*/*+* and *Psc^1^*/*+*; *mbf1^2^*/*+* to near wild-type levels (Fig. 2D).

In addition to the extra sex comb phenotype, *Psc^1^*/*+*; *mbf1^2^*/*+* exhibited misexpression of Ubx in wing discs (Fig. 2E, top). The signals appeared as spots consisting of clusters of Ubx-positive cells. The *pcm*^Δ*1*^ mutation decreased the number of spots per wing disc. The misexpression occurred predominantly around the dorsoventral border in the posterior compartment. Consistently, we observed adult wing defects along the posterior wing margin, which was also suppressed by *pcm*^Δ*1*^ (Fig. 2E, right).

### Mbf1 protects *E(z)* mRNA from Pcm activity

**Fig. 3.**
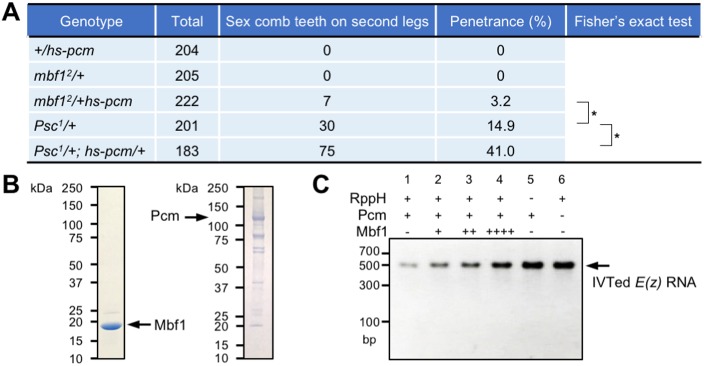
**Mbf1 protein directly counteracts 5′-3′ exoribonuclease activity *in vivo* and *in vitro*.** (A) *mbf1^2^/hs-pcm* double heterozygotes exhibit the Polycomb phenotype in the wild-type Polycomb group background. *P<0.01. (B) Recombinant *Drosophila* Mbf1 and Pcm preparations were resolved by 5-20% SDS-PAGE and the gel stained with Coomassie Brilliant Blue. (C) Recombinant *Drosophila* Mbf1 inhibits Pcm activity *in vitro*. *In vitro*-transcribed *E(z)* RNA [IVTed *E(z)* RNA] was used as substrate for Pcm. Reactions included the indicated components, and purified RNAs were resolved on a 1.5% agarose gel. dsDNA marker size is indicated (bp). Amounts of Mbf1 added: +, 2.5 µg; ++, 5 µg; ++++, 10 µg.

Importantly, we detected the extra sex comb phenotype under mild overexpression of *pcm* in *mbf1^2^*/*hs-pcm* double heterozygotes at 25°C, even in the wild-type Polycomb group background (Fig. 3A). *hs-pcm*/+ exhibited an ∼2.5-fold overexpression of Pcm at 25°C (Fig. S3A). Nevertheless, *hs-pcm* heterozygotes in the wild-type *mbf1* background did not show any Polycomb phenotype. These results suggest that Mbf1 stabilizes Polycomb silencing against fluctuations in the Pcm protein level *in vivo*. We also observed enhancement of the Polycomb phenotype in *Psc^1^*/+; *hs-pcm*/+ compared with that in *Psc^1^*/+ (Fig. 3A).

Biochemical analyses using purified recombinant Mbf1 and Pcm proteins (Fig. 3B) revealed that Mbf1 protects *E(z)* RNA from degradation by Pcm. RNA protection assays were performed in which *in vitro-*synthesized *E(z)* RNA was treated with the RNA pyrophosphatase RppH to convert the 5′-triphosphoryl end into the 5′-monophosphoryl form, which is a Pcm substrate. The RNA was digested with Pcm in the presence or absence of Mbf1. Mbf1 inhibited the digestion of *E(z)* RNA (Fig. 3C, lanes 2-4 versus lane 1). In the absence of RppH, RNA degradation was barely detectable (Fig. 3C, lane 5), suggesting that the digestion was due to 5′-exoribonuclease activity. Gel filtration of a mixture of Pcm and Mbf1 resulted in the elution of each protein in a clearly separated peak (Fig. S3B). Furthermore, Mbf1 did not co-immunoprecipitate with Pcm and vice versa (Fig. S3C). These results suggest that Mbf1 does not inhibit Pcm activity through protein-protein interactions. Collectively, we conclude that Mbf1 protects *E(z)* mRNA from degradation by Pcm both *in vivo* and *in vitro*.

### Model and implications of Mbf1 binding to mRNA

**Fig. 4.**
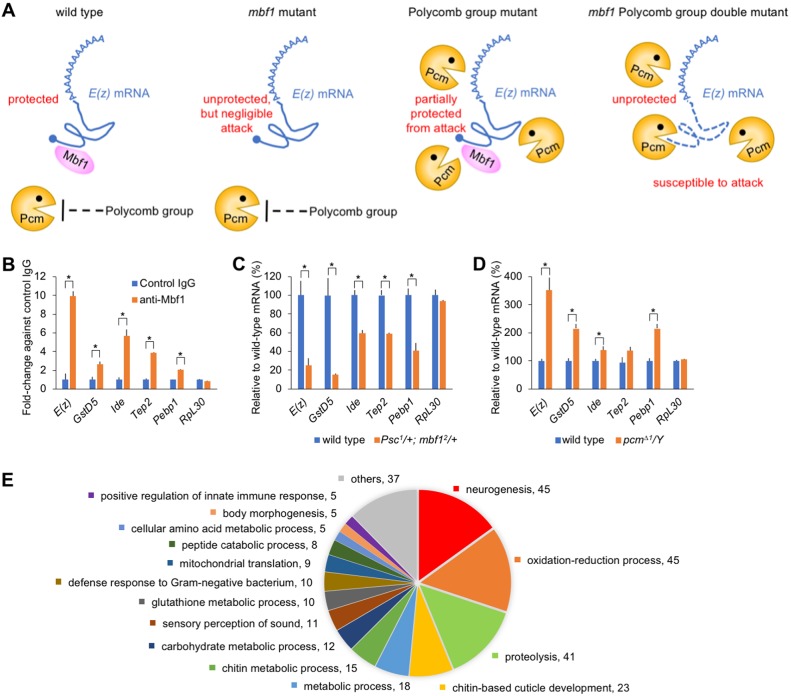
**Conceivable functions of cytoplasmic Mbf1 protein via binding to mRNAs.** (A) Model for Mbf1-ensured Polycomb silencing. In wild-type and *mbf1* mutant lines, Pcm is not upregulated. Therefore, the steady-state level of *E(z)* mRNA is well balanced irrespective of Mbf1 expression. In Polycomb group mutants, Pcm expression is upregulated so that *E(z)* mRNA could become susceptible to Pcm attack. However, Mbf1 protects *E(z)* mRNA to ensure robustness of Polycomb silencing. In *mbf1* and Polycomb group double mutants, loss of Mbf1 allows extensive degradation of *E(z)* mRNA by derepressed Pcm, thereby affecting Polycomb silencing. (B) The enrichment of four representative mRNAs (*GstD5*, *Ide*, *Tep2* and *Pebp1*) identified in the RIP-seq results was confirmed by RIP RT-qPCR analysis. Results for *E(z)* and *RpL30* mRNAs from Fig. 1F are included for comparison. Data are mean±s.d. of fold-change versus control IgG; *P<0.01. (C,D) RT-qPCR analysis of the indicated mRNAs in whole extracts of third instar male larvae from *Psc^1^/+*; *mbf1^2^/+* (C) or *pcm*^Δ*1*^*/Y* (D). Data are mean±s.d. relative to the wild-type mRNA level; *P<0.01. All *P*-values obtained using Student's *t*-test. (E) Gene ontology analysis of the RNA-seq results is consistent with the known Mbf1 functions. The number of genes in each term is indicated.

We propose that cytoplasmic Mbf1 ensures Polycomb silencing by protecting *E(z)* mRNA from the activity of Pcm (Fig. 4A). In the *mbf1* mutant, *E(z)* mRNA is free from Mbf1 protein, but *pcm* expression is downregulated by Polycomb group genes. In the Polycomb group mutant, Pcm expression is upregulated, but *E(z)* mRNA is partly protected by Mbf1. In the *mbf1* Polycomb group double mutant, *E(z)* mRNA is free from Mbf1 protein and is subject to Pcm attack. Whereas Mbf1 is highly expressed in undifferentiated cells, such as those of embryos, larval testis, ovary, imaginal discs and neuroblasts, its expression is reduced in differentiated tissues (Fig. S4A; see also Jindra et al., 2004), similar to the situation in the *mbf1* mutant. This would facilitate the rapid release of developmental genes from Polycomb silencing upon differentiation. Interestingly, expression of mammalian Mbf1 [also termed endothelial differentiation-related factor 1 (Edf1)] (Dragoni et al., 1998) and Ezh2 (Ezhkova et al., 2009) declines immediately after the onset of differentiation.

A recent study demonstrated that Pcm prevents apoptosis in imaginal discs and downregulates specific transcripts such as *hid* and *reaper* (Waldron et al., 2015). However, suppression of apoptosis did not rescue the lethality of a *pcm* null mutation at the early pupal stage. Therefore, there might be other targets of Pcm that are essential for early pupal development. The present study indicates that *E(z)* mRNA could be one such target.

The mRNA-binding activity of Mbf1 was selective, but might not be strictly specific to *E(z)* mRNA. Although Polycomb silencing is central to the developmental regulation of gene expression, there could be other mRNAs that bind to Mbf1 in a similar manner, thereby modulating another biological function. Therefore, we conducted RIP-seq analysis to identify Mbf1-bound mRNAs. To ensure robustness of our RIP-seq data, we compared our results independently with two publically available datasets (Fig. S5A) and identified 804 commonly enriched mRNAs (Table S1). Among these, the enrichment of four representative mRNAs (*GstD5*, *Ide*, *Tep2* and *Pebp1*) was confirmed by RIP RT-qPCR analyses (Fig. 4B). Interestingly, the expression levels of these four mRNAs decreased in *Psc^1^/+*; *mbf1^2^/+* and increased in *pcm*^Δ*1*^*/Y* compared with those in wild type (Fig. 4C,D), suggesting that the model (Fig. 4A) can be applied to a wider range of mRNAs than just *E(z)*. However, dependency on the Mbf1/Pcm antagonism appears to differ among the mRNAs.

Gene ontology and pathway analyses of the 804 genes revealed some interesting properties of the Mbf1-associated mRNAs (Fig. 4E, Fig. S5B, Tables S2 and S3). The gene ontology terms ‘glutathione metabolic process’, ‘oxidation-reduction process’ and ‘neurogenesis’, which includes *E(z)*, are consistent with the fact that we previously found defects in oxidative stress defense and axon guidance in the *mbf1* mutant (Liu et al., 2003; Jindra et al., 2004). Also of interest are the groups ‘positive regulation of innate immune response’ and ‘defense response to Gram-negative bacterium’, as *Arabidopsis* *MBF1* is involved in host defense against microbial infection (Suzuki et al., 2005; Kim et al., 2007). Moreover, pathway analysis of the enriched genes implicated Mbf1 in ‘drug metabolism’, as previously suggested for tamoxifen resistance (Mendes-Pereira et al., 2012). This raises an intriguing possibility that Mbf1 contributes to various types of stress defense, metabolic processes and neurogenesis as both a nuclear coactivator and as a cytoplasmic mRNA-stabilizing protein. Although *mbf1* null mutants are viable under laboratory conditions, evolutionary conservation of *mbf1* suggests that it has essential role(s) under real-world stress conditions.

## MATERIALS AND METHODS

### Fly lines

*yw;* *mbf1^2^* and *yw;* *P*{*Mbf1^+^*}*;* *mbf1^2^* have been described (Jindra et al., 2004). *pcm*^Δ*1*^/*FM7* (Lim et al., 2009) was a gift from Dr T. Kai (Osaka University, Japan). *pcm^5^* and *P*{*CaSpeR hs-pcm*} (Grima et al., 2008) (designated *hs-pcm* here) were from Dr S. F. Newbury (University of Sussex, Brighton, UK). w*; *E(z)^731^ FRT2A/TM6C* was from Dr J. Müller (Max Planck Institute of Biochemistry, Munich, Germany). *yw: UAS-GFP;* *hh-Gal4* and *yw;* *UAS-mbf1* were from Dr Q.-X. Liu (Shandong Agricultural University, China). *Psc^1^*/*CyO* and *Pc^6^*/*TM3* were obtained from The Kyoto Stock Center. *yw;* *UAS-mbf1^RNAi^* was from The Bloomington Stock Center. *Pc^6^*/*TM6B w*^+^ *GFP*, *Psc^1^*/*CyO GFP*, *yw;* *mbf1^2^ Pc^6^*/*TM6B w^+^ GFP*, *pcm*^Δ*1*^*;* *mbf1^2^*, *pcm*^Δ*1*^*;* *hs-pcm*, *pcm^5^;* *hs-pcm* and *yw;* *Psc^1^*/*CyO;* *mbf1^2^*/*TM6B* lines were established through appropriate crosses. The expression level of *pcm* in wild type is extremely low and approximately half that of leaky expression from the *hsp70* promoter at 25°C in *hs-pcm/+*. Therefore, the *pcm* mutation can be rescued in *pcm*^Δ*1*^*;* *hs-pcm/+* without any heat shock.

### Polycomb phenotype

Females of *yw*, *yw;* *mbf1^2^*, *yw;* *P*{*Mbf1^+^*}*;* *mbf1^2^*, *Psc^1^*/*CyO*, *yw;* *Psc^1^*/*CyO;* *mbf1^2^*/*TM6B*, *pcm*^Δ1^, *pcm*^Δ1^*;* *mbf1^2^*, *pcm*^Δ1^*;* *hs-pcm*, *pcm^5^*, *pcm^5^;* *hs-pcm* or *hs-pcm* were crossed with males of *Psc^1^*/*CyO GFP*, *Pc^6^*/*TM6B w^+^ GFP*, *yw;* *mbf1^2^ Pc^6^*/*TM6B w^+^ GFP*, *yw;* *mbf1^2^*, w*; *E(z)^731^ FRT2A/TM6C* or *hs-pcm*. After rearing at 25°C, male progeny of desired genotype were used for inspection of the Polycomb phenotype. As the Polycomb phenotype is significantly affected by rearing conditions, such as ingredients of the fly diet, the penetrance should be compared within the same experiment. Statistical analysis was performed by Fisher's exact test.

### Immunostaining

Rabbit polyclonal antiserum was raised against bacterially expressed polypeptides carrying the C-terminal region of Pc, the N-terminal region of E(z) or the N-terminal region of Pho. Immunoblot data using these antibodies are shown in Fig. S6. Immunostaining of imaginal discs was carried out as described previously (Liu et al., 2003). Antibodies were used at the following dilutions: anti-Pc (1:1000), anti-E(z) (1:1000), anti-Pho (1:1000), anti-Ubx (Developmental Studies Hybridoma Bank; 1:1000), anti-Mbf1 (Jindra et al., 2004; 1:500), goat anti-rabbit IgG or anti-mouse IgG Alexa488 (Molecular Probes, A72731 and A32723; 1:2000) and anti-rabbit IgG-Cy5 (Jackson ImmunoResearch, 111-225-144; 1:500). Images were acquired with an LSM510 META confocal microscope (Zeiss).

### RIP

Approximately 0.8 ml packed volume of *Drosophila* embryos (0-22 h after egg laying) from *yw* or *yw;* *mbf1^2^* was homogenized with 2 ml lysis buffer comprising 100 mM Na phosphate (pH 7.1), 10 mM NaCl, 3 mM MgCl_2_, 0.5% (v/v) NP-40, 0.5 mM DTT, 1× Protease Inhibitor Cocktail (Sigma-Aldrich), 0.5 mM PMSF and 0.5 units/ml porcine liver RNase inhibitor (Takara) in a Dounce homogenizer. The homogenate was centrifuged at 20,000 ***g*** for 10 min and the supernatant collected as the cytoplasmic fraction. After addition of 5 M NaCl to a final concentration of 360 mM, 1 ml of the cytoplasmic fraction was mixed with anti-Mbf1 antibody (Abcam, ab 174651)-loaded or rabbit IgG-loaded Dynabeads protein A (Life Technologies) at 4°C for 2 h. The beads were washed with PBS containing 0.1% (v/v) Tween 20 and the immunoprecipitated materials were dissolved in 0.2 ml 6 M guanidine-HCl in 0.4 M Tris-acetate/1 mM EDTA (pH 8.0). RNA was purified using a Direct-zol RNA MiniPrep Kit (Zymo Research) with a DNase I treatment step and quantitated by RT-qPCR (see below). Data are presented as fold-change compared with the IgG control experiment. Each mean±s.d. was calculated from qPCRs performed in triplicate. Statistical analysis was performed by Student's *t*-test.

The previous antibody against *Drosophila* Mbf1 (Jindra et al., 2004) did not precipitate any RNA. As Mbf1 binds to mRNA through its N-terminal region (Klass et al., 2013), the antibody might mask the RNA-binding region. Therefore, we used antibody ab174651 (Abcam), which was raised against a peptide carrying the C-terminal region (amino acids 98-148) of human MBF1 (EDF1) (Fig. S7A). It was able to immunoprecipitate *Drosophila* Mbf1 from the cytoplasmic fraction of embryos (Fig. S7B).

### RT-qPCR

Total RNA was prepared from ten heads of third instar male larvae, or a cytoplasmic or nuclear fraction from 60 wing discs of the desired genotype. GFP signals were used to exclude larvae with the GFP balancers. cDNAs were prepared from RNA samples of two biological replicates. qPCR was performed using a Roche LightCycler 2.0 as described previously (Nakayama et al., 2012) on each cDNA in three to five technical replicates. Primer sequences are listed in Table S4. Data were normalized by the *βTub56D* mRNA level and then presented as relative to the wild-type mRNA level. As the fluctuation between biological replicates did not differ substantially from that among technical replicates, the data (mean±s.d.) from a representative biological replicate are shown in figures. Statistical analysis was performed by Student's *t*-test.

### Western blot

Western blotting was performed as described previously (Nakayama et al., 2012) on samples containing 60 wing or leg discs. Antibodies were used at the following dilutions: anti-E(z) (1:4000), anti-Pc (1:4000), anti-Pho (1:4000), anti-Pcm (gift of S. F. Newbury; 1:2000), anti-Spt16 (Dre4) (Nakayama et al., 2012; 1:4000), anti-Mbf1 (1:5000), anti-FLAG M2 (Sigma, F3165; 1:2000), anti-tubulin (Developmental Studies Hybridoma Bank, a gift of K. Saito, National Institute of Genetics, Japan; 1:5000), anti-rabbit and anti-mouse IgG-HRP (GE Healthcare, NA9340 and NA9310; 1:5000) and anti-mouse IgG-HRP.

### RNA protection assay

*E(z)* RNA was *in vitro* transcribed from T7 vector template (a derivative of Drosophila Genomics Resource Center clone LD30505) using the MEGAscript Kit (Ambion). A reaction comprised 0.5 µg *E(z)* RNA, 2.5 units recombinant *E. coli* RNA 5′-pyrophosphohydrolase (RppH) (NEB), 0.5 µg baculovirus-expressed recombinant *Drosophila* Pcm protein, 5 units RNase inhibitor (Roche), 10 µg BSA and 0.1% Triton X-100 in 10 µl 1× NEB2 buffer (New England Biolabs), with the titrating amount of bacterially expressed *Drosophila* Mbf1 protein (2.5-10 µg). RNA was purified using a DNA Clean&Concentrator Kit (Zymo Research). An aliquot of the purified RNA was subject to agarose gel electrophoresis and stained with ethidium bromide.

### Purification of bacterially expressed recombinant proteins

*Drosophila* *mbf1* or mouse *Mbf1* cDNA was cloned into pET28a (EMD Biosciences) or pQE80L (Qiagen) vector, respectively. To prepare antigen for expression, *Drosophila* cDNA encoding the C-terminal region (amino acids 190-390) of Pc, the N-terminal region (amino acids 2-219) of E(z) or the N-terminal region (amino acids 2-302) of Pho was cloned into pQE80L. *E. coli* BL21(DE3) was transformed with each vector, and expression of recombinant proteins was induced by adding IPTG to 0.5 mM. Recombinant proteins were purified using Ni-NTA agarose (Qiagen) according to the supplier's protocol.

The recombinant Mbf1 protein was further purified by passing through a 30 kDa cut-off spin-concentrator and then concentrated on a 10 kDa cut-off spin-concentrator (both Amicon Ultra, Millipore) while exchanging the buffer to 50 mM Na phosphate (pH 7.0), 0.2 mM EDTA.

### Purification of baculovirus-expressed recombinant Pcm protein

Recombinant *Drosophila* Pcm protein (residues 1-1141) with the C-terminal FLAG tag was expressed as previously described (Jinek et al., 2011) using the Bac-to-Bac system (Invitrogen), and was purified using Ni-NTA agarose (Qiagen). The protein was concentrated on a 30 kDa cut-off spin-concentrator (Amicon Ultra, Millipore) while exchanging the buffer to 20 mM Tris-HCl (pH 8.3), 420 mM NaCl, 0.1% Triton X-100, 0.2 mM EDTA, 20% glycerol.

### Gel filtration analysis of Pcm and Mbf1 proteins

A Sephacryl S-200 HR (GE Healthcare) column (5 mm×185 mm) was prepared and equilibrated with 10 mM Tris-HCl (pH 8.0), 150 mM NaCl, 0.2 mM EDTA, 1 mM DTT. Input material consisted of 50 µg each of Pcm and Mbf1 in 30 µl. Fractions were collected of 50 µl after void fractions.

### Co-immunoprecipitation

Anti-FLAG M2 (Sigma), anti-Mbf1 or rabbit normal IgG were each conjugated to 10 µl Dynabeads Protein G (Dynal) in TBST (Tris-buffered saline pH 8.0 containing 0.1% Tween 20) and 5% skimmed milk overnight at 4°C, and the beads then washed extensively with TBST. Pcm (5 µg) and Mbf1 (5 µg) proteins were mixed in 200 µl TBST, and added to each bead preparation. The mixtures were rotated for 3 h at room temperature. After washing the beads with TBST five times, bound proteins were eluted in 100 mM Tris-HCl (pH 6.8) and 4% SDS. An aliquot of each eluate was subject to western blot.

### RNA-seq and data analysis

Immunoprecipitated RNA was subjected to a library generation protocol using the SENSE mRNA-Seq Library Prep Kit (Lexogen). The library was sequenced using an Illumina HiSeq 2500. Reads were mapped on the custom dm6 transcriptome with 3′-untranslated regions using TopHat v2.1.1 (Trapnell et al., 2009). Transcript abundance was quantified as fragments per kilobase of transcript per million fragments mapped (FPKM) values using Cufflinks v2.1.1 (Trapnell et al., 2010), and analyzed using Cuffdiff v2.1.1 (Trapnell et al., 2013). Enrichment scores were calculated in log_10_ transformation of the modified FPKM value ratio between immunoprecipitated sample and the means of two independent publically available transcriptome datasets using embryonic poly(A)^+^ RNAs [modENCODE datasets (http://data.modencode.org/?Organism=D.%20melanogaster) (IDs: 2019-2023) and GSE57517], in which all the FPKM values were modified by addition of 1 to minimize dispersion effect. Genes demonstrating log_10_ of modified FPKM value >1 were extracted from each dataset, and common genes between the datasets (804 genes; Fig. S5A, Table S1) were subjected to gene ontology and pathway analyses using DAVID (https://david.ncifcrf.gov).

## Supplementary Material

Supplementary information

Supplementary information
