# Supplementary material for: Mbf1 ensures Polycomb silencing by protecting E(z) mRNA from degradation by Pacman
Source: Development. 2018 Mar 1;145(5):dev162461. doi: 10.1242/dev.162461 (PMC5868998; doi:10.1242/dev.162461)
Supplement: Supplementary information [file develop-145-162461-s1.pdf]

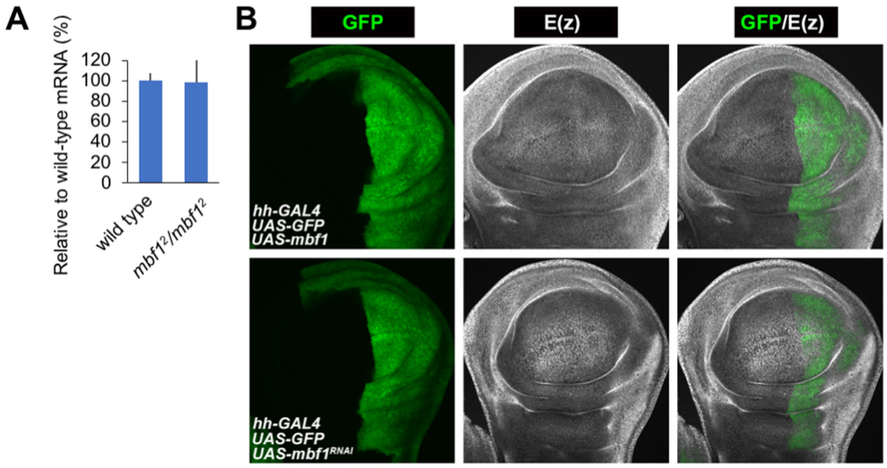

Figure S1. Fluctuation in expression level of *mbf1* does not influence on that of *E(z)*. (**A**) RT-qPCR analyses of *E(z)* mRNA levels in whole extracts from third instar male larvae. Data, mean ± s.d. of relative to the wild-type mRNA level. No significant difference was observed between wild type and *mbf1*^{2} /*mbf1*^{2}. (**B**) Immunofluorescence analyses of E(z) protein expression in wing discs of third instar larvae exhibiting no significant difference in the expression of E(z) protein upon overexpression (upper panels) or knockdown (lower panels) of Mbf1 protein. Mbf1 protein levels were under control of a GAL4-UAS system, and which coincided with GFP expression.

Genotype	Total	Sex comb teeth on second legs	Penetrance (%)	Fisher's exact test
Psc^{1}/+	312	44	14.1	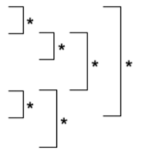
pcm^{5}; *Psc*^{1}/+	296	16	5.4	
pcm^{5}; *Psc*^{1}/+; *hs-pcm*/+	301	46	15.3	
pcm^{5}; *Psc*^{1}/+; *mbf1*^{2}/+	285	46	16.1	
pcm^{5}; *Psc*^{1}/+; *hs-pcm*+/+*mbf1*^{2}	212	70	33.0	
Psc^{1}/+; *mbf1*^{2}/+	298	90	10.1	

Figure S2. Another *pcm* allele *pcm*^{5} also suppresses the extra sex comb phenotype. *P < 0.01 by Fisher's exact test.

Development • Supplementary information

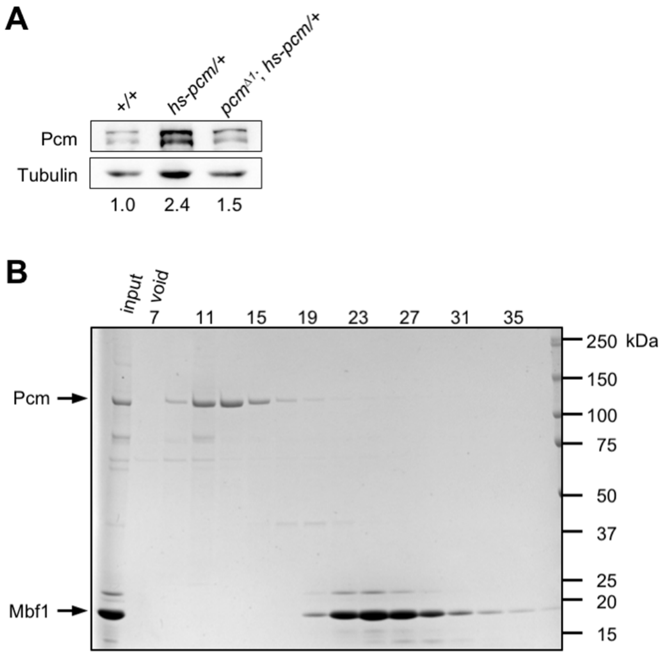

Figure S3. (**A**) Western blot analysis of Pcm in wing discs from indicated lines. Numbers denote the relative Pcm protein levels normalized with those of Tubulin. (**B**) Gel-filtration chromatography of a mixture of recombinant Pcm and Mbf1. Every odd-fractions starting from a void fraction were resolved on 5-20% SDS-PAGE and the gel was stained with Coomassie brilliant blue. (**C**) Immunoprecipitation experiments of Pcm and Mbf1 proteins. Anti-FLAG antibody and anti-Mbf1 antibody were used for immunoprecipitation and detection of FLAG-tagged Pcm protein and Mbf1 protein, respectively.

Development • Supplementary information

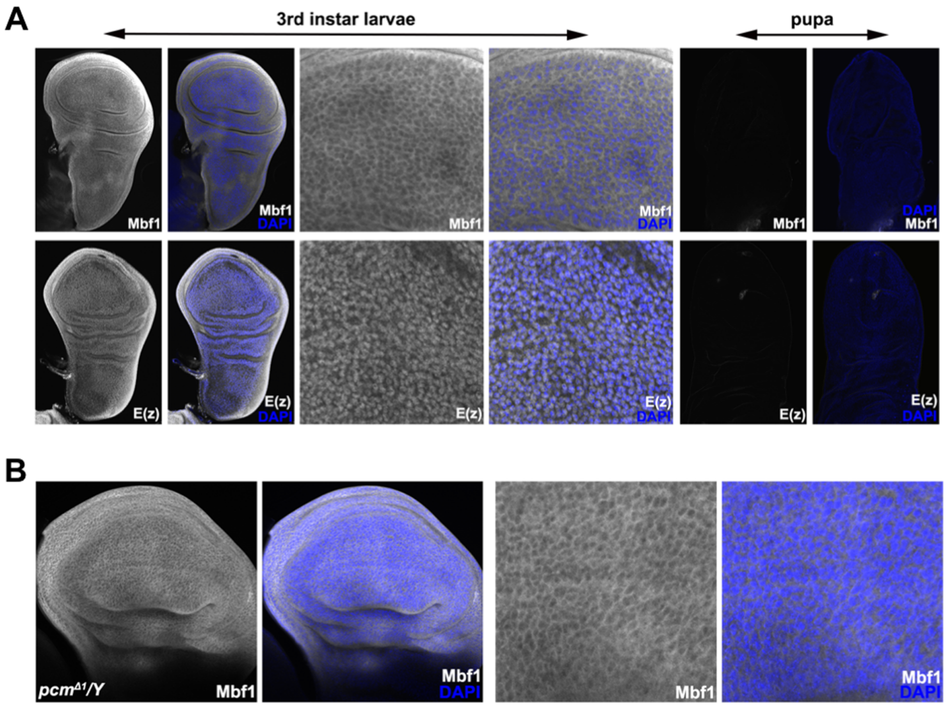

Figure S4. Immunofluorescence confocal microscopy analyses on expression and subcellular localization of Mbf1 and E(z) proteins. (**A**) Mbf1 and E(z) protein expression in wing discs of either third instar larvae or pupae. High-magnification views show that majorities of Mbf1 and E(z) proteins are detected in different cellular compartments in larvae (the right-side panels): Mbf1 in the cytoplasm and E(z) in the nuclei. However, both proteins are barely detectable in pupae. DNA was counterstained with DAPI (blue). (**B**) Mbf1 protein expression in a wing disc of $pcm^{\Delta 1}/Y$ third instar larva. Mbf1 protein is mainly detected in the cytoplasm also in the mutant, suggesting that Pcm protein exerts its effect on Polycomb silencing not through nuclear translocation of Mbf1. DNA was counterstained with DAPI (blue).

Development • Supplementary information

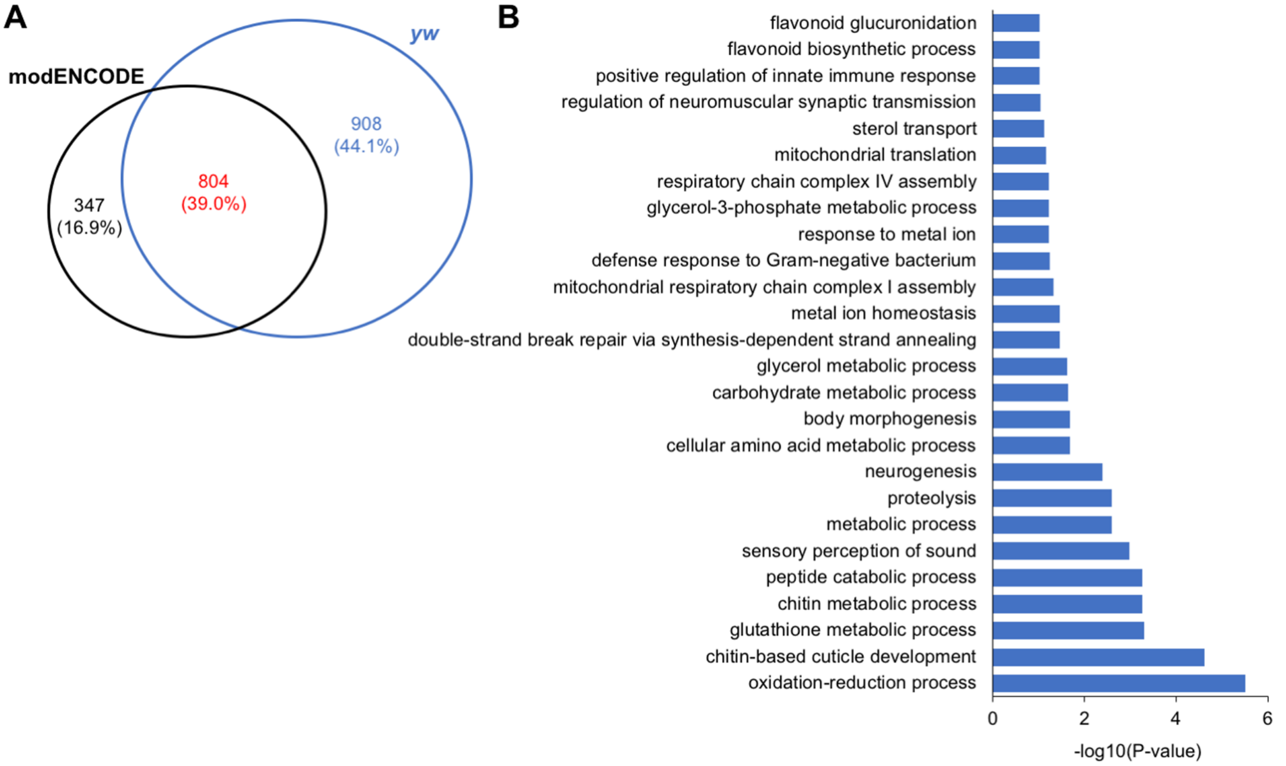

Figure S5. Results of RIP-seq analyses. (**A**) Venn diagram shows commonly enriched 804 genes between the datasets (69.9% of enriched genes from modENCODE datasets, 47.0% of those from *yw* datasets). (**B**) Gene-ontology analyses of the commonly enriched 804 genes. Each enriched biological process is represented by “-log10 (*P*-value)”. Details are shown in Table S2.

Development • Supplementary information

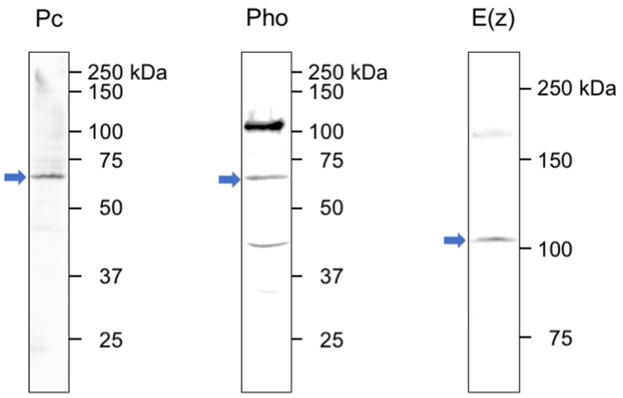

Figure S6. Immunoblot data for antisera against *Drosophila* Pc, Pho, and E(z). Embryonic nuclear extracts (0-22 hrs after egg laying, 25 µg each) were subjected to immunoblot analyses using each antiserum. Arrows indicated each target protein.

Development • Supplementary information

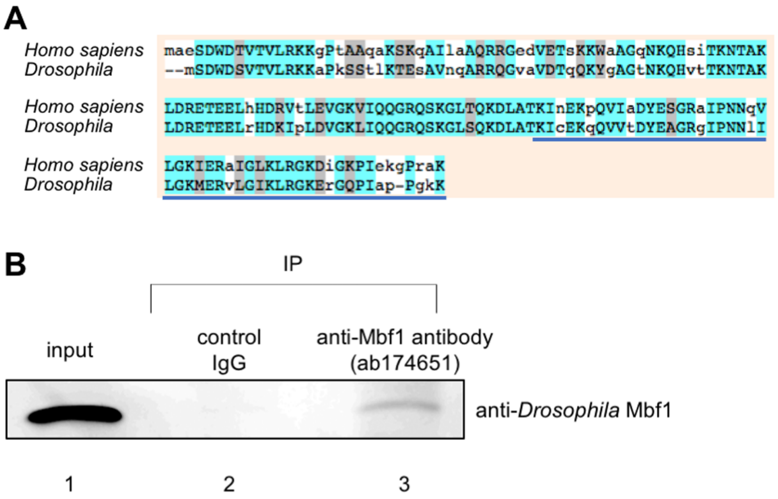

Figure S7. The anti-human Mbf1 antibody immunoprecipitates *Drosophila* Mbf1. (**A**) Amino acids sequence alignment between *Homo sapiens* and *Drosophila* Mbf1 using MUSCLE (Edgar, 2004). The underlined region was used as immunogen for generation of the anti-human Mbf1 antibody (abcam ab174651). (**B**) Either the anti-human Mbf1 antibody (ab174651) or control IgG was subjected to immunoprecipitation (IP) test using *yw* embryonic extracts. Lane 1, input; lane 2, IP by control IgG; lane 3, IP by ab174651. Detection, anti-*Drosophila* Mbf1 antibody.

Development • Supplementary information

Table S1. List of enriched genes in RIP-seq analysis

Click here to Download Table S1

Table S2. GO-term Biological Process

Click here to Download Table S2

Table S3. KEGG Pathway Analysis

Click here to Download Table S3

Table S4. Primers for RT-qPCR

Click here to Download Table S4
